# Regulation of GIP and GLP1 Receptor Cell Surface Expression by N-Glycosylation and Receptor Heteromerization

**DOI:** 10.1371/journal.pone.0032675

**Published:** 2012-03-07

**Authors:** Gina M. Whitaker, Francis C. Lynn, Christopher H. S. McIntosh, Eric A. Accili

**Affiliations:** 1 Cardiovascular Research Group, University of British Columbia, Vancouver, British Columbia, Canada; 2 Life Sciences Institute, Department of Cellular and Physiological Sciences, University of British Columbia, Vancouver, British Columbia, Canada; 3 Department of Surgery, University of British Columbia, Vancouver, British Columbia, Canada; 4 Diabetes Research Group, University of British Columbia, Vancouver, British Columbia, Canada; University of Ulster, United Kingdom

## Abstract

In response to a meal, Glucose-dependent Insulinotropic Polypeptide (GIP) and Glucagon-like Peptide-1 (GLP-1) are released from gut endocrine cells into the circulation and interact with their cognate G-protein coupled receptors (GPCRs). Receptor activation results in tissue-selective pleiotropic responses that include augmentation of glucose-induced insulin secretion from pancreatic beta cells. N-glycosylation and receptor oligomerization are co-translational processes that are thought to regulate the exit of functional GPCRs from the ER and their maintenance at the plasma membrane. Despite the importance of these regulatory processes, their impact on functional expression of GIP and GLP-1 receptors has not been well studied. Like many family B GPCRs, both the GIP and GLP-1 receptors possess a large extracellular N-terminus with multiple consensus sites for *Asn*-linked (N)-glycosylation. Here, we show that each of these *Asn* residues is glycosylated when either human receptor is expressed in Chinese hamster ovary cells. N-glycosylation enhances cell surface expression and function in parallel but exerts stronger control over the GIP receptor than the GLP-1 receptor. N-glycosylation mainly lengthens receptor half-life by reducing degradation in the endoplasmic reticulum. N-glycosylation is also required for expression of the GIP receptor at the plasma membrane and efficient GIP potentiation of glucose-induced insulin secretion from the INS-1 pancreatic beta cell line. Functional expression of a GIP receptor mutant lacking N-glycosylation is rescued by co-expressed wild type GLP1 receptor, which, together with data obtained using Bioluminescence Resonance Energy Transfer, suggests formation of a GIP-GLP1 receptor heteromer.

## Introduction

The hormones Glucose-dependent Insulinotropic Polypeptide (GIP) and Glucagon-like Peptide-1 (GLP-1) are released from gut endocrine cells into the circulation, in response to food ingestion. These peptide hormones act on specific G-protein coupled receptors (GPCRs), located in multiple tissues [1], [2], including the pancreatic β cell where both GIP and GLP-1 exert their actions by augmenting glucose-induced insulin secretion.

As for other intrinsic cell surface proteins and GPCRs [3], [4], the GIP and GLP-1 receptors (GIPR; GLP-1R) are synthesized in the rough endoplasmic reticulum and likely pass through various steps of post-translational modifications and quality control to ensure delivery of a correctly folded form to the cell surface. N-glycosylation is a key process that regulates exit of many GPCRs from the ER and delivery to the plasma membrane [4], [5], [6]. However, the influence of these processes on GIPR and GLP-1R expression and function has not been comprehensively studied.

Both GIPR and GLP-1R are expressed as glycoproteins in native tissues [7], [8], [9] implying that N-glycosylation plays a role in their function and/or cell surface expression. Indeed, treatment with tunicamycin, a fungicide that inhibits N-glycosylation, concentration-dependently reduced the number of GLP-1 binding sites and GLP-1-induced cAMP production in the RINm5F cell line, suggesting that N-glycosylation is important for functional surface expression [10]. The impact of N-glycosylation on GIPR surface expression or on GIP and GLP-1 potentiation of glucose-induced insulin secretion remains unexplored. Like all family B GPCRs, both GIPR and GLP-1R possess a large leucine-rich extracellular N-terminus with several potential sites for N-glycosylation [11], [12], but the extent to which each site is used and their individual impact on receptor function is not known.

Although able to function as monomers [13], [14], [15], GPCRs have been suggested to exist as homo- or hetero-oligomeric structures that influence cell surface expression and function [3], [5], [16]. However, whether oligomerization occurs among all GPCRs is unclear and has been intensely debated [5], [6], [17]. Studies using Bioluminescence Resonance Energy Transfer (BRET) support homomeric association of the GIPR [18] as well as heteromerization of the GLP1 and secretin receptors [19]. However, self-association of the GLP1R or close associations between the structurally-related GIPR and GLP1R have not been demonstrated; this is potentially critical given the overlap of GIPR and GLP1R expression and function in tissues such as the endocrine pancreas.

In this study, we examined N-glycosylation of the incretin receptors, GIPR and GLP-1R. To establish the extent to which each of the putative sites are N-glycosylated and their impact on function, we have carried out a mutational analysis of the N-terminus of the human GIPR and GLP-1R and examined cell signaling and surface expression using various approaches. Our data support a critical and, in the case of the GIPR, essential role for N-glycosylation in functional cell surface expression. Furthermore, we show that N-glycosylation is required for efficient GIP potentiation of glucose-induced insulin secretion from the pancreatic β-cell line, INS-1. Finally, we demonstrate that close associations of co-expressed GIPR and GLP1R occur, which act to restore functional expression of the GIPR that is otherwise abolished by the lack of N-glycosylation, suggesting the formation of receptor heteromers.

## Materials and Methods

### Plasmids and Mutagenesis

Human GLP1R cDNA was purchased from GeneCopoeia (OmicsLink Expression Clone EX-A0510-M02). Overlapping PCR mutagenesis was used to remove the stop codon and add common restriction sites in order to insert GLP1R cDNA into pcDNA3.1-V5 (Invitrogen Corp) such that the V5 epitope was expressed on the C terminal end of the receptor (GLP1R-V5). The human GIPR was purchased from Addgene (plasmid 14942, kindly donated by B Thorens) [20]. Overlapping PCR mutagenesis was used to remove the short form of the receptor and its stop codon from this plasmid as well as insert common restriction sites such that the cDNA could be inserted into pcDNA3.1-V5 with the V5 epitope expressed on the C terminal end of the receptor (GIPR-V5). For N-terminally HA-tagged constructs, overlapping PCR mutagenesis was used to insert the HA epitope (YPYDVPDYA) directly downstream of the GIPR and GLP1R putative signal peptide sequences on their N-termini. Quickchange PCR mutagenesis (Stratagene) was used to make all glycosylation point mutations in both V5-tagged and HA-tagged GIPR and GLP1R constructs. For BRET constructs, pGFP-N and pRLuc-N vectors (PerkinElmer), along with GIPR-V5 and GLP1R-V5 were digested using common restriction sites such that either GFP or Renilla Luciferase (RLuc) fusion proteins were expressed in frame on the C-termini of both receptors. All tagged constructs were tested for functionality using cAMP assays (data not shown). The ion channel HCN2-GFP was utilized as a control, having been tested functionally in a previous study [21].

### Cell Culture and Expression

CHO-KI cells (American Type Culture Collection) were maintained in Ham's F-12 medium (Invitrogen Corp) supplemented with 50 ug/ml penicillin/streptomycin (Invitrogen Corp) and 10% fetal bovine serum (Sigma-Aldrich) and incubated at 37°C with 5% CO_2_. After 24 h, once cells were approximately 80% confluent, they were transiently transfected with mammalian expression vectors encoding tagged wild type or mutant receptors using FuGene6 transfection reagent (Roche Diagnostics).

### Tunicamycin treatment

Three µg of cDNA were used for transfection in 50 mm dishes. Six hours after transfection, tunicamycin (5 µg/ml) (Santa Cruz Biotechnology Inc) or DMSO was added to cells, which were then incubated overnight. Cells were then lysed for 30 minutes on ice with radioimmunoprecipitation assay buffer (RIPA buffer: 50 mM Tris at pH 8.0, 1% Nonidet P-40, 150 mM NaCl, 1 mM EDTA, 1 mM PMSF, 2 mM each Na_3_VO_4_ and NaF, and 10 µg/ml each aprotinin, pepstatin, and leupeptin). 20 µg of protein was loaded in 10% SDS-PAGE gels.

### Cell Surface Biotinylation

Three µg of cDNA were used for transfections in 50 mm dishes. 24 hours after transfection, cells were washed twice with 1× PBS (pH 8.0), then 1 ml Versene (Invitrogen Corp) was added to detach cells. Cells were collected and washed twice with 1× PBS. 300 µl of 5 mM Sulfo-NHS-SS-Biotin (Thermo Scientific) was added to cells for 45 minutes at room temperature. Cells were washed with 1×PBS supplemented with 100 mM glycine to quench biotin reaction. Cells were then resuspended in RIPA buffer and lysed on ice for 30 minutes followed by centrifugation to collect supernatant. 100 µg of biotinylated lysate were added to 100 µl of 50% streptavidin-conjugated agarose beads (Sigma-Aldrich), and bead-lysate mix was incubated overnight at 4°C with rotation. Beads were washed with 1× PBS supplemented with 0.1% NP-40, and incubated with Laemmli sample buffer supplemented with 1 mM DTT for 1 hour at room temperature, then loaded in 10% SDS-PAGE gels.

### Western Blotting

Proteins separated on SDS-PAGE gels were transferred onto PVDF membranes using the i-Blot transfer device (Invitrogen Corp). Membranes were then blocked with 5% non-fat milk in TBST overnight at 4°C. Mouse-anti V5 (1∶1000, Invitrogen Corp) was added to the membrane and incubated for 2.5 hours at room temperature. Blots were washed three times in TBST, followed by addition of 1∶3000 anti-mouse secondary antibody conjugated to horseradish peroxidase (Santa Cruz Biotechnology Inc) for 1 hour at room temperature. Blots were washed in TBST, followed by chemiluminescence detection.

### ELISA Assays

Three µg of cDNA were used for transfections in 50 mm dishes. 24 hours after transfection, cells were seeded into 96-well assay plates (Corning Inc) at a density of approximately 100,000 cells per well. After 24 hours, cells were fixed with 4% paraformaldehyde and antigen detected with mouse-anti HA primary antibody (1∶1000, Sigma-Aldrich), followed by anti-mouse-HRP conjugated secondary antibody (1∶1000 Santa Cruz Biotechnology Inc) and SuperSignal ELISA Femto Maximum Sensitivity Substrate (Thermo Scientific). Signals were detected using the Victor 3 V plate reader (PerkinElmer). Each condition was carried out in triplicate, and data was collected from a minimum of 5 independent transfections. Mutants and wild type receptor data were compared using one-way ANOVA followed by Dunnett's multiple comparison tests (Graph Pad Prism).

### TR-FRET cAMP assays

For transfections in 50 mm dishes, 0.5 µg of cDNA was used. 24 hours after transfection, cells were rinsed with 1× HBSS (Invitrogen Corp), detached with 1 mL Versene (Invitrogen Corp), then resuspended at a density of 6000 cells per 5 µl in stimulation buffer, pH 7.4 (1× HBSS supplemented with 5 mM HEPES buffer, 0.1% BSA and 0.5 mM IBMX). Both GLP-1 7–36 amide and GIP peptides (Sigma-Aldrich) were diluted in stimulation buffer. Assay was then performed according to LANCE TR-FRET cAMP assay kit protocol (PerkinElmer), in 96-well white optiplates (PerkinElmer). Peptide stimulation of cells was carried out for 30 minutes and detection for 1 hour at room temperature, followed by fluorescence detection on a Victor 3 V plate reader (PerkinElmer), according to LANCE protocol parameters. RFU values generated at each dose were subtracted from values obtained using untransfected cells. Data for cAMP curves were fitted to the sigmoidal dose-response equation (Graph Pad Prism) and EC_50_ values for individual experiments were calculated. Each curve was generated from at least 5 independent transfections and EC_50_ values were compared to wild type using one-way ANOVA, followed by Dunnett's multiple comparison tests.

### Half-life Assay

Twenty-four hours after transfection of V5-tagged constructs, cycloheximide (Sigma-Aldrich) was added to cells in F-12 media with serum to a final concentration of 100 ug/ml. Cells were then lysed at times 0 h, 2 h, 4 h and 8 h after addition of cycloheximide. DMSO was added to transfected cells as a negative control. 30 ug of protein lysate were loaded into SDS-PAGE gels and western blots were performed as described.

### Immunocytochemistry, Imaging and Pearson Correlations

Forty-eight hours after transfection, CHO cells were rinsed with PBS and fixed with 2% paraformaldehyde in PBS for 5 min. Fixed cells were washed twice with PBS, permeabilized for 10 min using 0.2% Triton X-100, then blocked with 10% normal goat serum (NGS) for 10 min. After one wash with PBS containing 1% NGS, cells were incubated with primary antibodies for 1 h at room temperature. Anti-HA (Sigma-Aldrich) mouse monoclonal antibody was used at a dilution of 1∶500 and rabbit anti-calnexin (Sigma-Aldrich) at a dilution of 1∶100. Cells were subsequently washed with PBS three times and incubated with Alexa-555-tagged anti-mouse and Alexa-488-tagged anti-rabbit secondary antibodies (Molecular Probes, Inc.) at a dilution of 1∶1500 in PBS with 1% NGS for 1 h at room temperature in the dark. After washing three times in PBS, coverslips were rinsed in H_2_O and mounted on slides using Gel Mount (Sigma-Aldrich). Cells were visualized using a Zeiss Axiovert 200 fluorescence microscopy with an Apotome structured illumination module and with a ×63 oil immersion objective lens. Results reported represent three transfections for each set of the imaging experiments described. To correlate intensities of fluorescence for each pair of proteins, Pearson correlation coefficients were calculated from captured images of individual cells as determined by the following equation (Axiovision User Guide),
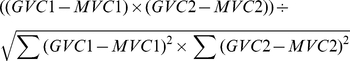
where *GV* represents Gray Value, *MV* is Mean Value, and *C* is channel.

The values range from −1 to +1, representing an increasing correlation of the intensities measured in two channels. In other words, the Pearson correlation coefficient describes the interdependence of varying intensities of fluorescence between two proteins of interest throughout a cell.

### Culture of INS-1 Cells

INS-1 (clone 831/12) cells were obtained from Dr. C.B. Newgard (Duke University) [22]. Cell lines were maintained at 37°C with 5% CO_2_. Cells were grown in RPMI-1640 medium containing 11 mM glucose, supplemented with 10% fetal bovine serum (Cansera, Rexdale ON), and penicillin/streptomycin, supplemented with 10 mM HEPES (pH 7.4), 1 mM sodium pyruvate, 2 mM glutamine and 50 µM β-mercaptoethanol.

### Saturation Binding Analysis in INS-1 Cells

INS-1 cells were plated in 24 well plates at a density of 5×10^5^ cells/well and allowed to grow for 24 hours, then treated with 1 µg/ml tunicamycin for 24 hours. Saturation binding experiments were carried out as previously described [23]. Data were analyzed using a one site model for GIP binding and then fitted to a curve with the equation:
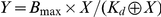
where B_max_ is the binding obtained when cells are saturated with ^125^I-GIP, and Kd is the concentration of ^125^I-GIP required to reach half-maximal binding. The number of receptors on each cell was determined using the specific activity of the radiolabel and Avogadro's number.

### Insulin Release from INS-1 Cells

Cells were plated into 24 well plates (5×10^5^ cells/well) and grown for 24 hours. Cells were treated with 1 µg/ml tunicamycin for 24 hours, followed by incubation with either 5.5 mM or 11 mM glucose with or without 50 nM GIP for 30 minutes at 37°C, then assayed for insulin content using RIA. The means were compared using two-tailed ANOVA followed by Dunnett's multiple comparison tests.

### BRET^2^ assays

Here, 0.5 µg of RLuc-tagged construct and 0.5–3 µg of GFP-tagged construct cDNA were used for transfections in 50 mm dishes. BRET^2^ experimental methods were carried out according to our previously published protocol [24]. 24 hours after transfection, cells were rinsed with DPBS (Invitrogen Corp) and treated with 1 mL of 0.5% trypsin-EDTA (Invitrogen Corp). 3 ml of HAM's F12 media containing FBS was added; cells were centrifuged at 800×g and resuspended in BRET^2^ Buffer (DPBS supplemented with 2 µg/ml Aprotinin). Approximately 100,000 cells were distributed into 96-well white optiplates (PerkinElmer). Using a Victor 3 V plate reader (PerkinElmer), expression of GFP-tagged constructs was assessed by directly exciting GFP with a 400–410 nm excitation filter. Expression of Rluc-tagged constructs was assessed using luminescence values obtained in the BRET^2^ assay. For BRET^2^ measurements, DeepBlueC substrate (PerkinElmer) was added to the cells at a final concentration of 5 µM, and Rluc emission was measured through a 370–450 nm filter. Resulting GFP emission was in turn measured with a 500–530 nm filter. All raw data were corrected by subtracting the BRET^2^ ratio and GFP/Rluc values determined from cells transfected with only RLuc-tagged construct (plotted at 0,0). Data were fitted to a single binding site equation by non-linear regression (GraphPad Prism).

## Results

### Multiple N-glycosylation consensus sites are present and utilized on the N-termini of the human GIPR and GLP-1R

**Figure 1:**
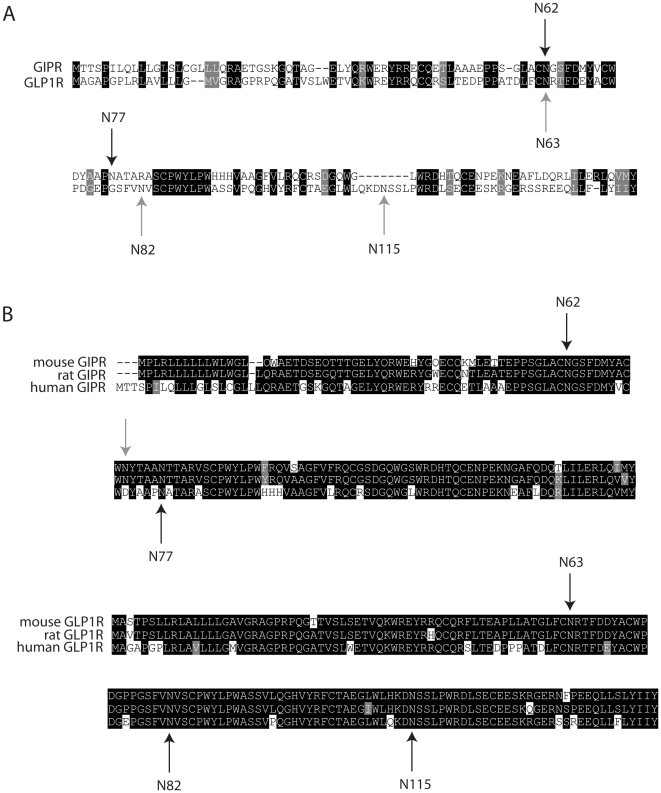
Consensus sites for N-glycosylation are found in the N-termini of the closely related human GIPR and GLP-1R. The amino acid sequences for human GIPR and GLP-1R (**A**) and three mammalian species for each receptor (**B**) were aligned by *ClustalW. Shading* was carried out using *Boxshade* on the Swiss EMBnet node web site (www.ch.embnet.org/software/BOX_form.html). Amino acids highlighted in *black* represent complete identities, whereas those highlighted in *gray* represent conserved identities. Arrows are placed above or below putative N-glycosylation sites and the numbers correspond to their location in the human sequences. In “A”, the black and gray arrows correspond to the *Asn* (N) residues in the human GIPR and GLP-1R, respectively. In “B”, the gray arrow corresponds to an *Asn* site found in the rodent sequences but not the human sequence.

The human GIPR and GLP-1R possess large N-terminal domains containing 2 and 3 putative N-glycosylation sites, respectively, as predicted by the NXS/T consensus sequences for N-glycosylation where X≠Proline. The locations of the two putative sites in the GIPR correspond closely to those of the GLP-1R (Fig. 1A), suggesting some evolutionary conservation of N-glycosylation between them. The human GLP-1R has an additional N-glycosylation site (N115), the closest to the first transmembrane domain, which appears as an insertion when aligned with the GIPR sequence. As for the human GLP-1R, the mouse and rat GIPR have three putative N-glycosylation sites on their N termini (Fig. 1B). However, the location of the third site in the rodent GIPR does not correspond to that in the GLP-1R, but is instead found between the two conserved sites (N62 and N77). Here, we focus on N-glycosylation of sites within the N-termini of the human GIPR and GLP-1R.

**Figure 2:**
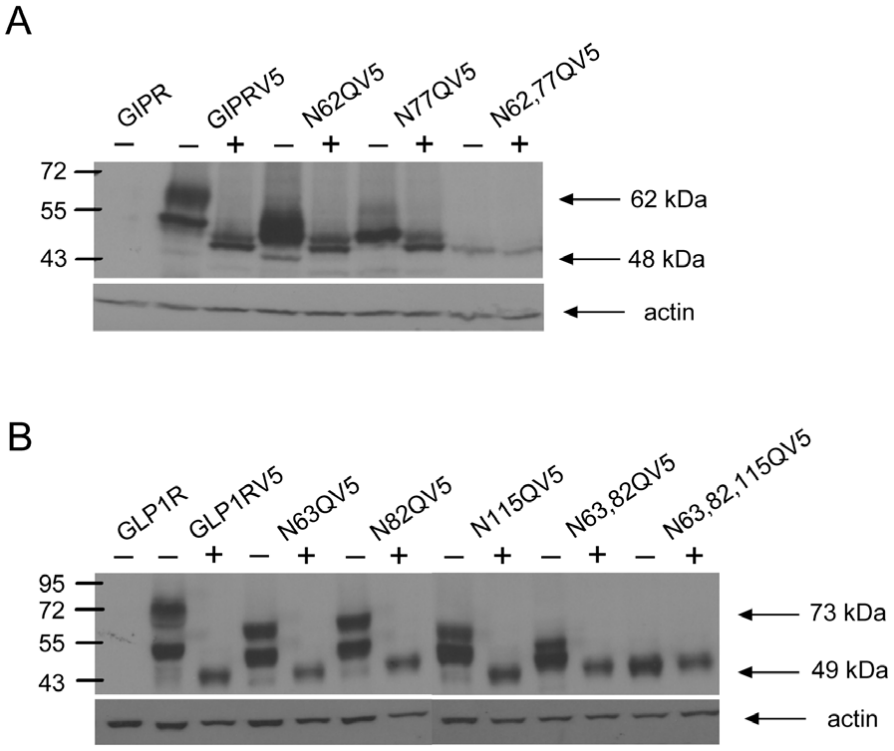
All putative N- glycosylation sites found on the N-termini of human GIPR and GLP-1R are used. Western Blots of C-terminally V5-tagged GIPR (**A**) and GLP-1R (**B**) and respective tagged mutant receptors in which putative N-glycosylation sites were substituted by glutamine, as indicated. Blots were probed with mouse monoclonal antibody against the V5 epitope. For both receptors, blots were also probed with a rabbit polyclonal antibody for actin to control for loading. Lanes marked with “+” were treated with tunicamycin, and “−” with DMSO control. Untagged GIPR and GLP-1R are used as negative controls for non-specific antibody binding. Upper and lower arrows indicate the molecular weights for the heaviest bands and lightest bands. The predicted molecular weight for the immature form of GIPRV5 is 57.7 kDa and of GLP-1RV5 is 57.4 kDa.

To determine whether each of the putative N-glycosylation sites is utilized, we individually and progressively mutated all sites in the human GIPR and GLP-1R from *Asn* to *Gln* and performed western blotting to determine whether corresponding shifts in molecular weight were produced. Both wild type receptors migrated at higher molecular weights than when treated with tunicamycin, an antibiotic that selectively inhibits oligosaccharyltransferase to block the addition of N-acetylglucosamine onto dolicho-phosphate (Fig. 2A,B). Furthermore, mutation of all putative N-glycosylation sites yielded single bands that migrated at lower weights, unaltered by tunicamycin. The molecular weights of receptors with single site mutations were reduced compared to wild type GIPR and GLP-1R, but were still greater than those following treatment with tunicamycin. Together, these data suggest that all putative sites in both receptors are glycosylated when expressed in CHO cells.

Two bands migrating at different weights can be seen in the absence of tunicamycin for most constructs, likely representing the presence of both core and complex glycosylated species. A second band of higher molecular weight is present in tunicamycin-treated GIPRs, and is also present in N62,77Q-GIPR lanes on blots with longer exposure times (data not shown), suggesting the presence of a second unglycosylated form. Treatment of lysates with PNGase, which cleaves sugar moieties from *Asn* residues, produced band patterns identical to those using tunicamycin (data not shown), as expected.

### N-glycosylation promotes cell surface expression of GLP-1R and is required for surface expression of GIPR

**Figure 3:**
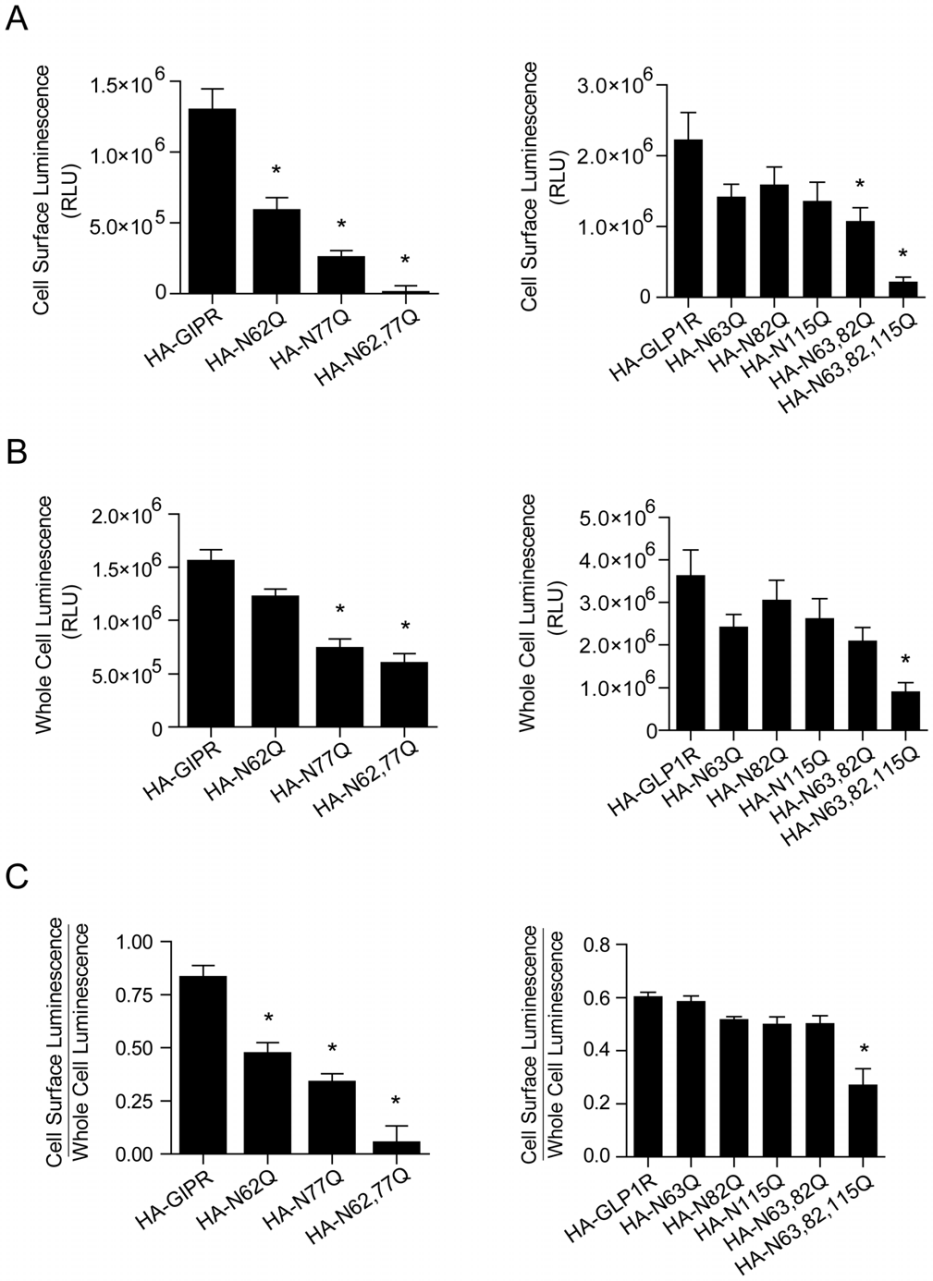
N-glycosylation more strongly impacts cell surface expression of the GIPR than the GLP-1R. Plots of cell surface luminescence (**A**), whole cell luminescence (**B**) and their ratio (**C**) from cells expressing N-terminally HA-tagged GIPR (left) and GLP-1R (right). All constructs were assayed in triplicate from 4–5 separate transfections. Each group of triplicates was corrected for background luminescence from untransfected cells and from cells treated with only secondary HRP-conjugated antibody for each transfected condition. Asterisks represent significance (P<0.05) as determined using a one-way ANOVA, followed by Dunnett's multiple comparison tests, comparing mutant constructs to tagged wild type receptors.

To examine the influence of N-glycosylation on cell surface expression, GIPR and GLP-1R were quantified at the plasma membrane by ELISA, using externally HA-tagged receptor constructs. When individual sites were mutated, a significant decrease in GIPR but not in GLP-1R cell surface expression was observed when compared to wild type receptors (Fig. 3A). With all N-glycosylation sites missing, surface expression of GLP-1R was still detected, whereas GIPR cell surface expression was virtually abolished. This trend was maintained when cell surface protein was calculated as a fraction of whole cell protein (Fig. 3C). Thus, the relative reduction in mutant surface protein expression was not predominantly a result of the observed decreases in intracellular protein (Fig. 3B).

**Figure 4:**
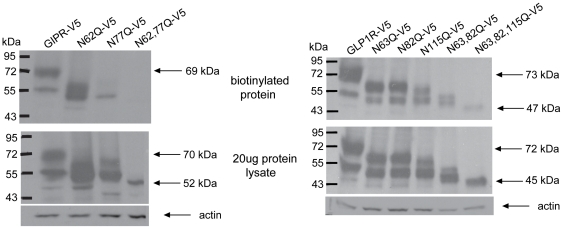
N-glycosylation increases the presence of cell surface isoforms of GIPR and GLP-1R protein. Western Blots of biotinylated cell surface protein (upper) and total protein lysate (middle) for V5-tagged GIPR (left) and GLP-1R (right). Upper and middle blots were probed with a mouse monoclonal antibody to the V5 epitope while the lower blots were probed with a rabbit polyclonal antibody to actin as a loading control. Note the absence of biotinylated protein in the lane containing N62,77Q-GIPR but not in the lane containing N63,82,115Q-GLP-1R.

To complement our findings from cell surface ELISA, we examined receptor surface protein using biotinylation assays. Not surprisingly, the intensities of the bands in western blots for both receptors at the cell surface and total lysates showed patterns that reflected those obtained by cell surface ELISA (Fig. 4). For both GIPR and GLP-1R lacking any N-glycosylation sites, the total protein (Fig. 4, lower protein lysates) was somewhat reduced whereas the cell surface protein (Fig. 4, upper blot) was more strongly decreased (N63,82,115Q-GLP-1RV5) or absent (N62,77Q-GIPRV5). Interestingly, there was a more pronounced reduction in amount of cell surface protein for N77Q-GIPR when compared to the N62Q-GIPR, again consistent with the findings from ELISA (Fig. 3A).

The blots of biotinylated protein also reveal multiple forms of wild type incretin receptors at the cell surface, likely representing variably N-glycosylated proteins. Single and double mutations of the GLP-1R, as well as the N62Q-GIPR, were also found at the cell surface in multiple forms. In contrast, only one immature form was observed for the N77Q-GIPR and the GLP-1R lacking all N-glycosylation sites.

### Sensitivity and efficacy of the cAMP response of the GIPR and GLP-1R to their natural ligands are augmented by N-glycosylation at a single site

**Figure 5:**
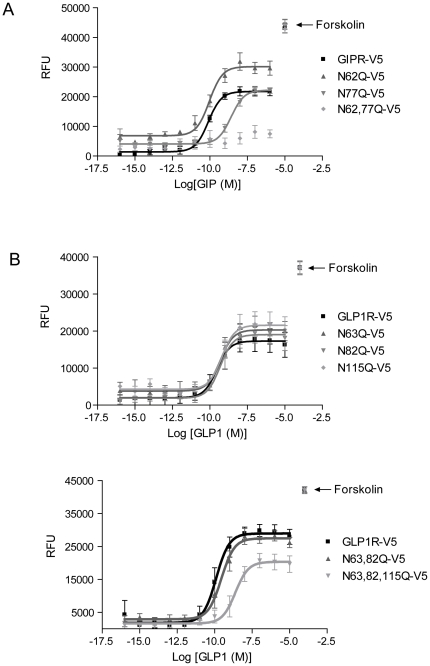
Sensitivity and efficacy of GIP and GLP-1 actions on cAMP production in CHO cells are enhanced by N-glycosylation of their receptors. Plots of Relative Fluorescence Units (RFU) versus log of concentration of GIP (**A**) or GLP-1 (**B**). RFU values were determined by FRET assay which was carried out using CHO cells expressing GIPR or GLP-1R constructs as indicated. Forskolin was used to control for variations in total cell number. Mean data ± SE (performed in duplicate for an n = 5 separate transfections) at each ligand concentration were plotted, converted to a logarithmic scale, and fitted using a sigmoidal dose-response curve.

To investigate the role of N-glycosylation in receptor function, cAMP production was measured in CHO cells expressing GIPR or GLP-1R using a FRET-based assay. For both wild type receptors, increases in cAMP levels were observed in response to increasing concentrations of their respective ligands (GIP or GLP-1; Fig. 5). Elimination of all receptor N-glycosylated sites abolished cAMP production by GIP but not by GLP-1. This is not surprising given the observed absence of GIPR but not GLP-1R at the cell surface.

In all instances, with the exception of N77Q-GIPR, removal of a single glycosylation site on either receptor resulted in concentration-response curves similar to those of corresponding wild type receptors (Fig. 5A and B, upper). Furthermore, N63,82Q-GLP-1R displayed concentration-response curves that did not differ from wild type GLP-1R (Fig. 5B, lower). In contrast, the EC_50_ values for N63,82,115Q-GLP-1R and N77Q-GIPR were significantly greater than those determined for the wild type receptors (Table 1). The amplitude of the cAMP response was also reduced for N63,82,115Q-GLP-1R.

**Table 1:** Mean EC_50_ values and amplitude of responses for cAMP concentration-response curves in Figure 5.

A.						
	*EC_50_ (M)*			*Amplitude (RFU)*		
	*Mean*	*S.E.*	*P Value*	*Mean*	*S.E.*	*P Value*
GIPR-V5	8.2×10^−11^	1.7×10^−11^	-	2.2×10^4^	1.3×10^3^	-
N62Q-V5	1.0×10^−10^	1.5×10^−11^	P>0.05	2.7×10^4^	3.3×10^3^	P>0.05
N77Q-V5	3.1×10^−9^	1.1×10^−9^	P<0.01	2.2×10^4^	6.5×10^2^	P>0.05
N62,N77Q	-	-	-	8.0×10^3^	1.4×10^3^	P<0.01

B.						
	*EC_50_ (M)*			*Amplitude RFU*		
	*Mean*	*S.E.*	*P Value*	*Mean*	*S.E.*	*P Value*
GLP1R-V5	4.1×10^−10^	1.2×10^−10^	-	1.7×10^4^	3.7×10^3^	-
N63Q-V5	6.3×10^−10^	1.4×10^−10^	P>0.05	1.9×10^4^	3.9×10^3^	P>0.05
N82Q-V5	7.1×10^−10^	2.6×10^−10^	P>0.05	1.9×10^4^	2.6×10^3^	P>0.05
N115Q-V5	7.2×10^−10^	1.1×10^−10^	P>0.05	2.0×10^4^	4.2×10^3^	P>0.05

	*EC_50_ (M)*			*Amplitude RFU*		
	*Mean*	*S.E.*	*P Value*	*Mean*	*S.E.*	*P Value*
GLP1R-V5	2.2×10^−10^	1.1×10^−10^	-	2.9×10^4^	9.0×10^2^	-
N63,82Q-V5	5.8×10^−10^	2.9×10^−10^	P>0.05	2.8×10^4^	1.9×10^3^	P>0.05
N63,82,115Q-V5	2.7×10^−9^	6.8×10^−10^	P<0.01	2.0×10^4^	2.0×10^3^	P<0.01

EC_50_ values were determined for each individual experiment, using a sigmoidal dose-response equation. Compared to wild-type receptors, the EC_50_ values for N77Q-GIPR and N63,82,115Q-GLP-1R were significantly right shifted, as shown by a one-way ANOVA analysis followed by Dunnett's multiple comparison test. The changes in RFU values for the N62,77Q-GIPR were small and could not be accurately fitted. The amplitude of response was calculated by subtracting the lowest RFU values from the highest for each individual experiment.

The rightward shift in concentration-response curves is observed in the same two mutants that displayed the lowest protein expression on the cell surface (see Fig. 3), suggesting a connection between the quantity of receptors expressed at the surface and the sensitivity of the cAMP response. The rightward shift could, however, be reflective of impaired function, and/or coupling to adenylyl cyclase, of misfolded surface-localized receptors.

The right-shift of the N77Q-GIPR, but not the N62Q-GIPR concentration-response curve suggests that the former site exerts functional dominance. Interestingly, cell surface ELISA data also suggests that site N77 plays a stronger role in regulating cell surface expression (see Fig. 3). In other intrinsic membrane proteins of the plasma membrane, non-uniformity among multiple sites has been noted [25], [26], [27].

### N-glycosylation of incretin receptors prevents degradation in the endoplasmic reticulum

**Figure 6:**
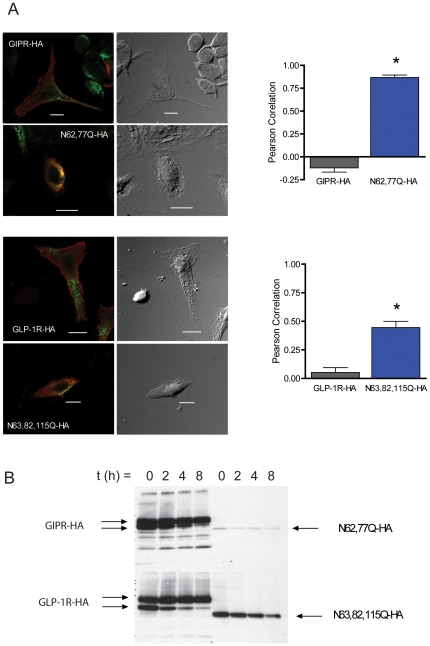
N-glycosylation-deficient incretin receptors are retained in the endoplasmic reticulum and degraded to a greater extent than wild type. (**A**) Co-localization of both wild type and N-glycosylation-deficient receptors with calnexin shown in photographs (left); the correlation of their intensities is shown in the bar graphs (right). (**B**) Western blots showing wild type and N-glycosylation-deficient incretin receptors at indicated time points following cycloheximide administration to CHO cells expressing each receptor.

First, the amount of receptor protein that associated with calnexin in the endoplasmic reticulum was quantified, by correlating the intensity of receptor-calnexin co-immunolabelling (Fig. 6A). For the GLP receptors, the wild type showed a low level of correlation whereas for the complete glycosylation-deficient mutant the correlation was much higher, at just under 50% (Fig. 6A, lower). For the GIP receptor, an even greater contrast was observed between the wild type receptor, which showed almost no correlation, and the complete glycosylation-deficient mutant in which correlation is about 80% (Fig. 6A, upper) Together, the data suggest that the reduction in glycosylation- deficient mutants at the cell surface is mainly due to an increase in ER retention and degradation. These data are also consistent with a larger role for N-glycosylation of the GIP receptor than for the GLP-1 receptor.

To address whether mis-folding and ER degradation is reduced by N-glycosylation, we used western blotting to determine the stability of the glycosylation deficient receptors following cycloheximide application. The rate of disappearance of glycosylation deficient receptors over time was increased compared to that of wild type receptors (Fig. 6B). Moreover, even at time = 0, the overall level of glycosylation-deficient receptors is much lower than that of wild-type receptors; this is especially apparent for the GIP mutant receptor.

### N-glycosylation maintains cell surface GIP receptor number and GIP-potentiated insulin secretion in INS-1 cells

**Figure 7:**
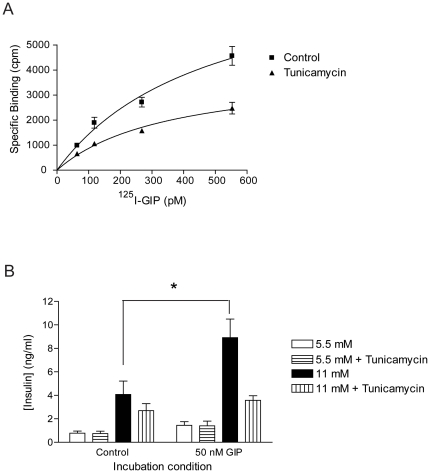
N-glycosylation regulates GIP receptor number and GIP-potentiated insulin secretion in INS-1 cells. (**A**) Plot of specific binding versus concentration of ^125^I-GIP saturation carried out in INS-1 cells with or without 1 µg/ml tunicamycin. Data were fitted with a non-linear regression equation assuming a single binding site (GraphPad Prism). Control cells expressed an average of 2443±400 GIP receptors on the cell surface (Kd 455±50 pM), whereas tunicamycin-treated cells expressed 760±70 GIP receptors on the cell surface (Kd 345±100 pM). (**B**) Plot of insulin release in response to glucose and/or GIP, from INS-1 cells with or without 1 µg/ml tunicamycin. Data are expressed as mean ± SEM, n = 4 separate experiments. Asterisk indicates a significant increase in insulin release compared to basal conditions without addition of 50 nM GIP (P<0.05), as determined using two-way ANOVA followed by Bonferroni post-tests.

We next examined the role of GIPR N-glycosylation in regulating ligand binding, surface receptor number and insulin secretion. We chose the INS-1 β-cell line, which reflects a similar physiology to that of native β-cells, including GIP-induced potentiation of insulin secretion in the presence of glucose [22]. INS-1 cells were treated with tunicamycin and specific binding of GIP was measured using a radioactive binding assay. Tunicamycin lowered cell surface ^125^I-GIP binding, which, at saturating levels, corresponded to a 70% decrease in cell surface GIPR number (Fig. 7A). The dissociation constants (Kd) of GIP from the surface of these cells did not significantly differ between the control (455±50 pM) and tunicamycin (345±100 pM) treated cells, suggesting that ligand binding affinity was not impaired by removal of N-glycosylation.

To investigate the impact of N-glycosylation on GIPR incretin response, GIP-stimulated insulin secretion was measured in tunicamycin-treated and untreated INS-1 cells. Without tunicamycin, GIP (50 nM) significantly and strongly potentiated insulin secretion, when increasing glucose from 5.5 mM to 11 mM (Fig. 7B). Tunicamycin treatment, however, blunted the incretin effect of GIP. Tunicamycin did not significantly modify insulin secretion in response to glucose alone, suggesting that GIP potentiation was eliminated specifically by the inhibition of N-glycosylation. These results are consistent with a reduction in GIP receptor number, but do not discount a potential impairment in GIPR function and/or signal transduction by the deficiency in N-glycosylation.

### The function and cell surface expression of a GIPR mutant completely lacking N-glycosylation is rescued by close association with the wild type GLP1R

**Figure 8:**
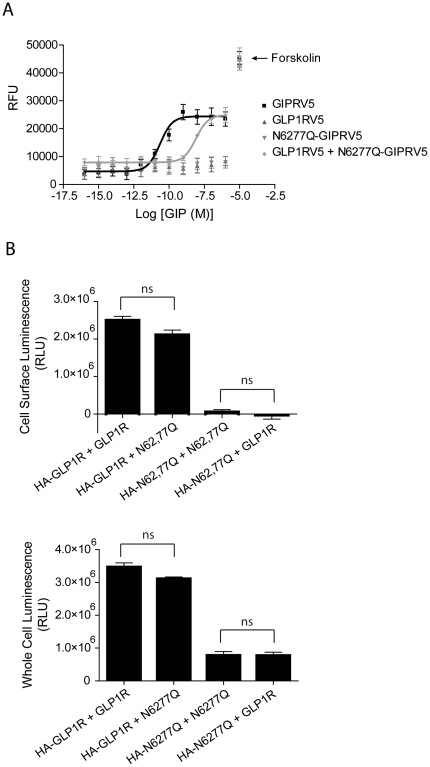
Function but not cell surface expression of N62,77Q-GIPR is rescued by co-expression of wild type GLP1R. (**A**) Plots of Relative Fluorescence Units (RFU, indicative of cAMP production) versus log of concentration of GIP. RFU values were determined by FRET assay which was carried out using cells expressing GIPR and GLP1R as indicated. Forskolin was used to control for variations in total cell number. Mean data ± SE (n = 5 transfections) at each ligand concentration were fit using a sigmoidal dose-response curve. (**B**) Plots of cell surface (left) and whole cell (right) luminescence from cells co-expressing N-terminally HA-tagged GIPR and/or GLP1R, as indicated. All constructs were assayed in triplicate from 4–5 separate transfections. Each group of triplicates was corrected for background luminescence from untransfected cells and from cells treated with only secondary HRP-conjugated antibody for each transfected condition. Pairs of receptors (grouped in brackets), consisting of one HA-tagged construct and a second untagged construct, were statistically compared by a two-tailed unpaired t-test (p>0.05 for each comparison made).

It has been suggested that many GPCRs exist as homo- or heterodimers and/or higher order oligomers at the cell surface and, in some cases, this interaction may be initiated during translation [28], [29]. Because the GIPR and GLP1R have a relatively high level of sequence identity (Fig. 1) and have similar expression profiles and physiological function, we reasoned that the GLP1R might form a functional complex with the GIPR. To test this, we first examined whether cell surface expression and function of the GIPR mutant lacking N-glycosylation could be rescued by co-expression with wild type GLP1R. Cyclic AMP levels were again measured in CHO cells expressing each receptor construct. In cells expressing N62,77Q-GIPR only, the amount of cAMP was unchanged from baseline in response to GIP. In contrast, the levels of cAMP increased in a concentration-dependent manner by GIP in cells co-expressing N62,77Q-GIPR along with the wild type GLP1R, although the EC_50_ value was right shifted compared to wild type GIPR (Fig. 8A, Table 2). These results show that functional expression of N62,77Q-GIPR was rescued by co-expressed wild type GLP1R.

**Table 2:** Mean EC_50_ values and amplitude of response for cAMP concentration-response curves in Figure 8A.

	*EC_50_ (M)*			*Amplitude RFU*		
	*Mean*	*S.E.*	*P Value*	*Mean*	*S.E.*	*P Value*
GIPR-V5	3.4×10^−11^	1.1×10^−11^	-	2.3×10^4^	2.8×10^3^	-
N62,N77Q-V5	-	-	-	5.0×10^3^	7.1×10^2^	P<0.01
GLP1R-V5	-	-	-	6.3×10^3^	2.0×10^3^	P<0.01
GLP1R-V5+N62,N77Q-V5	1.1×10^−8^	5.2×10^−9^	P<0.0001	1.9×10^4^	2.3×10^3^	P>0.05

EC_50_ values were determined for each individual experiment, using a sigmoidal concentration-response equation. Compared to wild-type GIPR, the EC_50_ values for N62,77Q-GIPR when co-expressed with the wild type GLP1R was significantly right shifted, as shown by a one-way ANOVA analysis followed by Dunnett's multiple comparison test. The changes in RFU values for N62,77Q-GIPR or the GLP1R were small and could not be accurately fitted. The amplitude of response was calculated by subtracting the lowest RFU values from the highest for each individual experiment.

In an attempt to quantify the extent of N62,77Q-GIPR cell surface rescue by wild type GLP1R, cell surface ELISA was again used. When the HA-tagged mutant GIPR was co-expressed with wild type GLP1R, the measured cell surface luminescence was very low and not significantly altered as compared to co-expression with the untagged version of itself (Fig. 8B, upper). Since the amplitude of cAMP responses to GIP were identical for both wild type GIPR, and mutant GIPR in the presence of GLP1R, it appears that rescue of a very small amount of the N62,77Q-GIPR, below the level of detection by cell surface ELISA, suffices to fully restore activation of adenylate cyclase in the CHO cell system.

The rescue of only a small fraction of N62,77Q-GIPR to the cell surface could be explained by a corresponding retention of wild type GLP1R by the GIPR mutant. However, this was not the case since the amount of HA-tagged GLP1R at the cell surface was unchanged by co-expression with N62,77Q-GIPR (Fig. 8B, upper). Moreover, when co-expressed, the total level of mutant GIPR and wild type GLP1R were no different than when each was co-expressed with non-HA tagged versions (Fig. 8B, lower). Therefore, it seems more likely that functional rescue of the mutant GIPR is due to a low level of association with the wild type GLP1R, which is below the detection sensitivity of the ELISA assay.

**Figure 9:**
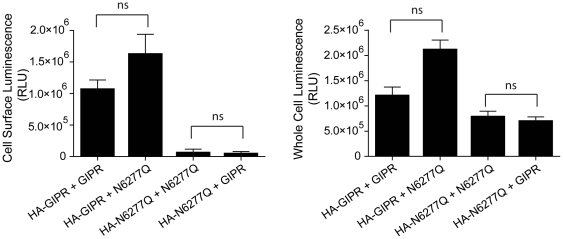
Cell surface expression of N62,77Q-GIPR is not rescued by co-expression with the wild type GIPR. Plots of cell surface (left) and whole cell (right) luminescence from cells co-expressing HA-tagged GIPR as indicated. All constructs were assayed in triplicate from 4–5 separate transfections. Each group of triplicates was corrected for background luminescence from untransfected cells and from cells treated with only secondary HRP-conjugated antibody for each transfected condition. Pairs of receptors (grouped in brackets), consisting of one HA-tagged construct and a second untagged construct, were statistically compared by a two-tailed unpaired t-test (p>0.05 for each comparison made).

The low level of association between N62,77Q-GIPR and wild type GLP1R could be due to the small differences in their primary sequences, thus potentially limiting interaction between key regions. To test this, we co-expressed N62,77Q-GIPR with wild type GIPR and measured cell surface and total protein expression of both receptor constructs by ELISA. Co-expression of wild type GIPR did not rescue cell surface expression of its mutant counterpart, nor was its own level at the cell surface altered (Fig. 9, left). Thus, the extent of receptor homology is not likely a factor for limiting the amount of cell surface rescue of the mutant GIPR.

**Figure 10:**
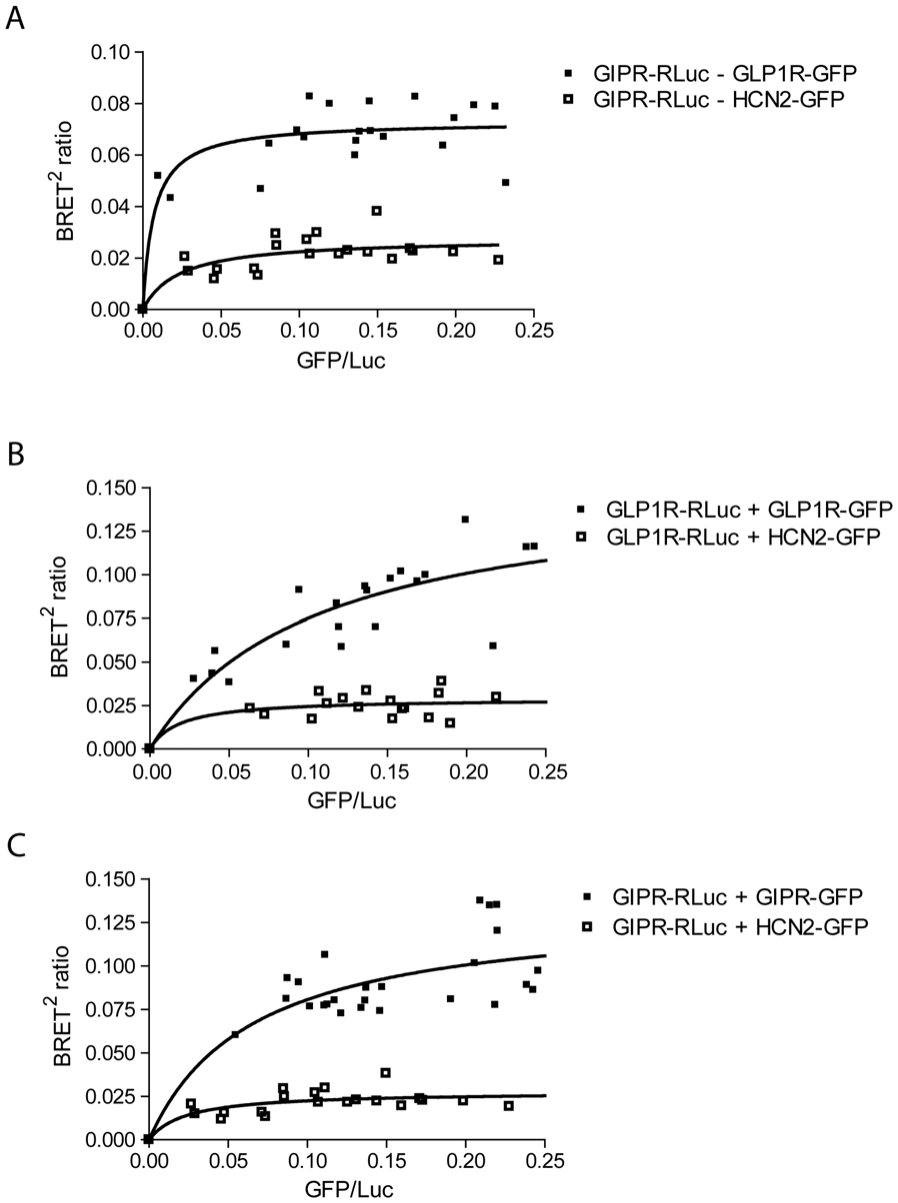
BRET values for cells expressing GIPR and GLP1R suggest heteromeric and homomeric associations. Plots of BRET^2^ ratio versus the ratio of GFP/Luc from cells co-expressing RLuc- and GFP-tagged combinations of GIPR, GLP1R and HCN2 channels. (**A**) Curves from GIPR+GLP1R (Bmax = 0.073±0.003) compared to GIPR+HCN2 negative control (Bmax = 0.028±0.004). (**B**) Curves from GLP1R+GLP1R (Bmax = 0.15±0.03), compared to GLP1R+HCN2 negative control (Bmax = 0.029±0.006). (**C**) Curves from GIPR+GIPR (Bmax = 0.13±0.02), compared to GIPR+HCN2 negative control (Bmax = 0.0*28±0.004*). Data were collected from at least separate 5 transfections and fit with a non-linear regression equation assuming a single binding site; they were statistically analyzed using a two-taile*d u*npaired t test and for all conditions, Bmax was significantly greater than negative controls, p<0.001.

The functional rescue of the N62,77Q-GIPR by the wild type GLP1R nevertheless suggests some form of physical association. To test for this, we utilized BRET to measure the effects of co-expressing GFP-labeled forms of GLP1R with R*Luc*-tagged GIPR (Fig. 10A). For a negative control, we used GFP-tagged hyperpolarization-activated channel (HCN2-GFP), a structurally similar membrane protein that has been shown to localize to the cell surface and function normally when expressed in CHO cells [21], but which would not be expected to assemble with either receptor. We also tested the effects of co-expressing GFP- and R*Luc*- labeled GLP1R (Fig. 10B), as well as GFP- and R*Luc*- labeled GIPR (Fig. 10C). For all three receptor combinations, the BRET saturation curves reached higher maximal values than their respective negative controls. The high values of BRET obtained, coupled with the rescue of N62,77Q-GIPR function by the wild type GLP1R, support heteromeric and homomeric associations of the GIPR and GLP1R.

## Discussion

Upon translation of the GIPR or GLP-1R nascent chains in the endoplasmic reticulum, they become subject to glycosylation at the *Asn* residues located in the extracellular N-terminus. We have shown that each of the putative *Asn* residues is glycosylated when either the human GIPR or GLP-1R is expressed in CHO cells. For both receptors, cell surface expression and/or function were impaired by removal of N-glycosylation, although the GIPR was more strongly affected and possessed a single site (N77) that was dominant. Our results from ELISA experiments suggest that the decrease in cell surface receptor expression can be attributed to factors independent of variations in total expression. Our data also show enhanced ER retention and degredation in the absence of N-glycosylation, in parallel with disrupted cell surface expression. Our findings come from expression of CHO cells, which is a limitation as primary beta-cells are very difficult to transfect and the low level of GIP and GLP-1 expression makes studies on endogenous glycosylation very difficult.

Importantly, we found that N-glycosylation regulates GIPR cell surface expression and the potentiation of glucose-induced insulin secretion by nanomolar levels of GIP in INS-1 cells. These data are similar to those obtained with the GLP-1R in RINm5F cells, where cell surface receptor number and GLP-1-induced cAMP production are reduced by tunicamycin treatment [10]. Thus, N-glycosylation maintains a level of GIPR and GLP-1R surface expression that is critical for downstream signaling and regulation of insulin secretion. Notably, 30% of the normal total number of GIP receptors remained after tunicamycin treatment despite an absence of GIP-potentiation of glucose-induced insulin secretion. This implies that a threshold number of GIP receptors exist, which must be surpassed in order to potentiate insulin secretion to a level that could be measured by our assay. Our findings in CHO cells suggest that the reduction in cell surface expression in the absence of N-glycosylation in the INS-1 cells is also due to enhanced retention and degredation of GIPR in the endoplasmic reticulum. Basic mechanisms of cell surface trafficking and ER quality control are conserved among most cells [30], [31], but studies in beta cells are required to determine their precise impact on incretin receptor cell surface expression and lifespan.

When cAMP was assayed in response to increasing concentrations of ligand, cells containing glycosylation-deficient GLP-1R or N77Q-GIPR exhibited a right-shifted concentration-response curve. This could have reflected a direct defect in GIP or GLP-1 binding to their respective receptors, or to a coupling problem with the signal transduction machinery. However, our data of GIP-binding to INS-1 cells, and previous data on GLP-1 binding to RINm5F cells [10], showed that binding affinity was unaffected by tunicamycin treatment, whereas the total surface receptor number was significantly decreased along with potentiation of insulin secretion. These data are consistent with those in other studies of bradykinin B_2_, P_2_Y_12_ ADP, and the type 1α metabotropic glutamate receptors, which also found that ligand-binding affinity was unaltered by inhibition of N-glycosylation [32], [33], [34]. Thus, the reduction in incretin sensitivity that we observed is probably due impaired function, and/or coupling to adenylyl cyclase, of misfolded surface-localized receptors. Alternatively, since cell surface expression and, in the case of the glycosylation-deficient GLP-1R, the maximum cAMP response were also reduced in concert with sensitivity, it is possible that the efficiency of coupling to downstream effector molecules such as adenylyl cyclase correlates with cell surface receptor number. Such a relationship between EC_50_ and cell surface number has been reported for the β_2_-adrenergic receptor, but the mechanism underlying this association remains unknown [35], [36]. In these studies, the β_2_-adrenergic receptor was localized to caveolae to a lesser extent when cell surface number was reduced; this suggests that efficient signal transduction requires localization of receptors to these structures, which contain the necessary machinery for cAMP production.

The rescue of mutant GIPR function by the wild type GLP1R, and the close association between the two receptors as measured by BRET, suggests that they are able to associate in a receptor complex. According to a recent definition, receptor heteromers are “composed of at least two functional receptor units with biochemical properties that are demonstrably different from those of its individual components” [37]. Functional co-assembly is supported by reduced sensitivity of the rescued GIPR mutant to GIP when compared to the wild type GIPR, as seen by the right shift in EC_50_ for cAMP formation; however, this right shift could also be explained by a corresponding reduction in number of mutant receptors at the cell surface, compared to wild type. Therefore, it remains uncertain as to whether oligomerization of GIPR and GLP1R produces a receptor complex with unique properties and, thus, whether a GIP-GLP1 receptor heteromer exists in our system. We also found that oligomerization may have been limited between glycosylation-deficient GIPR and the wild type GLP1R, based on the lack of cell surface rescue. Indeed, various studies have shown that when N-glycosylation is removed, regular receptor dimer formation and stability is impaired, thereby impacting the receptor heteromer's ability to express at the cell surface and/or correctly function [38], [39], [40], [41], [42]. Studies that further examine the functional and structural nature of the association between GLP-1R and GIPR will be required to determine the complete nature of this association and whether a true receptor heteromer is formed by them.

Regulation of GIPR and GLP-1R by N-glycosylation may have important implications for type 2 diabetes (T2DM). There is a reduced incretin effect in human T2DM patients [43], which has been attributed to reduced ß-cell responsiveness to GIP [44] and, to a lesser extent, GLP-1 [45]. Moreover, reduced incretin receptor expression has been observed in animal models of T2DM [2]. It will be important to determine if post-translational modifications such as N-glycosylation are impaired in Type II diabetes, and whether this results in a reduction in cell surface GIPR and GLP-1R number along with the decreased incretin response of the ß-cell. The finding that heteromerization between GIPR and GLP1R can occur suggests that the much milder reduction in GLP1 responsiveness could be a direct consequence of the more profound and fundamental observed decrease in GIPR expression. This possibility could explain the restoration of responses to both incretins in diabetic patients in whom glucose was almost normalized by insulin treatment [45]. Direct evidence for heteromerization of GIPR and GLP1R in tissues such as the pancreas would be an essential next step in determining the potential physiological consequences, as well as the therapeutic implications of such an interaction.
